# Supplementary material for: HIV Tat controls RNA Polymerase II and the epigenetic landscape to transcriptionally reprogram target immune cells
Source: eLife. 2015 Oct 21;4:e08955. doi: 10.7554/eLife.08955 (PMC4733046; doi:10.7554/eLife.08955)
Supplement: Supplementary file 1. — DOI: http://dx.doi.org/10.7554/eLife.08955.041 [file elife-08955-supp1.docx]

**Supplementary file 1A. Compilation of 62 publicly available datasets (containing genes differentially expressed during HIV infection) used in the enrichment analysis.** See also **Supplementary** **file 1B** and **Figure 1–figure supplement 10A** for the data generated using these datasets.

| **Gene set** | **PMID** | **Reference** | **Comment** |
| --- | --- | --- | --- |
| BIOCARTA_HIVNEF_PATHWAY | 16199517 | http://www.broadinstitute.org/gsea/msigdb/cards/BIOCARTA_HIVNEF_PATHWAY | MsigDB c2.cp (canonical pathway) |
| GSE24081_CONTROLLER_VS_PROGRESSOR_HIV_SPECIFIC_CD8_TCELL_UP | 16199517 | http://www.broadinstitute.org/gsea/msigdb/cards/GSE24081_CONTROLLER_VS_PROGRESSOR_HIV_SPECIFIC_CD8_TCELL_DN | MsigdB c7 (Immuno sigs) |
| GSE24081_CONTROLLER_VS_PROGRESSOR_HIV_SPECIFIC_CD8_TCELL_UP | 16199517 | http://www.broadinstitute.org/gsea/msigdb/cards/GSE24081_CONTROLLER_VS_PROGRESSOR_HIV_SPECIFIC_CD8_TCELL_UP | MsigdB c7 (Immuno sigs) |
| PID_HIV_NEF_PATHWAY | 16199517 | http://www.broadinstitute.org/gsea/msigdb/cards/PID_HIV_NEF_PATHWAY | MsigDB c2.cp (canonical pathway) |
| REACTOME_ABORTIVE_ELONGATION_OF_HIV1_TRANSCRIPT_IN_THE_ABSENCE_OF_TAT | 16199517 | http://www.broadinstitute.org/gsea/msigdb/cards/REACTOME_ABORTIVE_ELONGATION_OF_HIV1_TRANSCRIPT_IN_THE_ABSENCE_OF_TAT | MsigDB c2.cp (canonical pathway) |
| REACTOME_APOBEC3G_MEDIATED_RESISTANCE_TO_HIV1_INFECTION | 16199517 | http://www.broadinstitute.org/gsea/msigdb/cards/REACTOME_APOBEC3G_MEDIATED_RESISTANCE_TO_HIV1_INFECTION | MsigDB c2.cp (canonical pathway) |
| REACTOME_BINDING_AND_ENTRY_OF_HIV_VIRION | 16199517 | http://www.broadinstitute.org/gsea/msigdb/cards/REACTOME_BINDING_AND_ENTRY_OF_HIV_VIRION | MsigDB c2.cp (canonical pathway) |
| REACTOME_EARLY_PHASE_OF_HIV_LIFE_CYCLE | 16199517 | http://www.broadinstitute.org/gsea/msigdb/cards/REACTOME_EARLY_PHASE_OF_HIV_LIFE_CYCLE | MsigDB c2.cp (canonical pathway) |
| REACTOME_FORMATION_OF_THE_HIV1_EARLY_ELONGATION_COMPLEX | 16199517 | http://www.broadinstitute.org/gsea/msigdb/cards/REACTOME_FORMATION_OF_THE_HIV1_EARLY_ELONGATION_COMPLEX | MsigDB c2.cp (canonical pathway) |
| REACTOME_HIV_INFECTION | 16199517 | http://www.broadinstitute.org/gsea/msigdb/cards/REACTOME_HIV_INFECTION | MsigDB c2.cp (canonical pathway) |
| REACTOME_HIV_LIFE_CYCLE | 16199517 | http://www.broadinstitute.org/gsea/msigdb/cards/REACTOME_HIV_LIFE_CYCLE | MsigDB c2.cp (canonical pathway) |
| REACTOME_HOST_INTERACTIONS_OF_HIV_FACTORS | 16199517 | http://www.broadinstitute.org/gsea/msigdb/cards/REACTOME_HOST_INTERACTIONS_OF_HIV_FACTORS | MsigDB c2.cp (canonical pathway) |
| REACTOME_LATE_PHASE_OF_HIV_LIFE_CYCLE | 16199517 | http://www.broadinstitute.org/gsea/msigdb/cards/REACTOME_LATE_PHASE_OF_HIV_LIFE_CYCLE | MsigDB c2.cp (canonical pathway) |
| REACTOME_ROLE_OF_NEF_IN_HIV1_REPL_AND_DISEASE_PATHO | 16199517 | http://www.broadinstitute.org/gsea/msigdb/cards/REACTOME_THE_ROLE_OF_NEF_IN_HIV1_REPLICATION_AND_DISEASE_PATHOGENESIS | MsigDB c2.cp (canonical pathway) |
| Nakamura_2012_HIV-1_LTR_regulation_by_PAAF1 | 22316138 | http://www.retrovirology.com/content/9/1/13 | HIV1 LTR regulation by PAAF1 |
| Massanella_2013_DEGs_before_vs_after_ART_UP | 23933117 | http://www.ncbi.nlm.nih.gov/pubmed/23933117 | Before vs. after ART up |
| Massanella_2013_DEGs_before_vs_after_ART_DN | 23933117 | http://www.ncbi.nlm.nih.gov/pubmed/23933117 | Before vs. after ART down |
| Krishnan_2004_Cellular_GeneX_in_HIV-Infected_Cell_Lines | 15308739 | http://jvi.asm.org/content/78/17/9458.short | HIV1 reactivation after latency |
| Martinez-Marino_2007_DEGs_S0042682206008877_table1 | 17240414 | http://www.sciencedirect.com/science/article/pii/S0042682206008877 | Suppression of HIV1 replication in CD8+ cells |
| Imbeault_2009_HIV-1_induced_IFN-1_response_in_CD4+Tc_8h | 19146679 | http://www.retrovirology.com/content/6/1/5 | HIV1 induced IFN response after 8h |
| Imbeault_2009_HIV-1_induced_IFN-1_response_in_CD4+Tc_24h | 19146679 | http://www.retrovirology.com/content/6/1/5 | HIV1 induced IFN response after 24h |
| Park_2007_HIV-1_NC_Overexpression_in_HEK293T | 18051367 | http://www.ncbi.nlm.nih.gov/pubmed/18051367 | Gene expression after Nucleocapsid overexpression |
| Park_2007_HIV-1_pTat_Overexpression_in_HEK293T | 18051367 | http://www.ncbi.nlm.nih.gov/pubmed/18051367 | Gene expression pTat overexpression |
| Wen_2005_HIV-1_infection_induced_changes_in_U937_HUT78_T-cells | 15885842 | http://www.ncbi.nlm.nih.gov/pubmed/15885842 | HIV1 induced change in U937 and HUT78 cells |
| Munier_etal_Retrovir2005_HIV-1_Latency_U1_and_ACH-2_UP | 16305739 | http://www.retrovirology.com/content/2/1/73 | HIV1 latency: infected U1 and ACH-2 cells up |
| Munier_etal_Retrovir2005_HIV-1_Latency_U1_UP | 16305739 | http://www.retrovirology.com/content/2/1/73 | HIV1 latency: infected U1 cells up |
| Munier_etal_Retrovir2005_HIV-1_Latency_ACH-2_UP | 16305739 | http://www.retrovirology.com/content/2/1/73 | HIV1 latency: infected ACH-2 cells up |
| Munier_etal_Retrovir2005_HIV-1_Latency_U1_and_ACH-2_DN | 16305739 | http://www.retrovirology.com/content/2/1/73 | HIV1 latency: infected U1 and ACH-2 cells down |
| Munier_etal_Retrovir2005_HIV-1_Latency_U1_DN | 16305739 | http://www.retrovirology.com/content/2/1/73 | HIV1 latency: infected U1 cells down |
| Munier_etal_Retrovir2005_HIV-1_Latency_ACH-2_DN | 16305739 | http://www.retrovirology.com/content/2/1/73 | HIV1 latency: infected ACH-2 cells down |
| Giri_etal_JLB2006_HIV_Bcell | 16940334 | http://www.jleukbio.org/content/80/5/1031.long | Cell specific gene sets |
| Giri_etal_JLB2006_HIV_CD4_Tcells | 16940334 | http://www.jleukbio.org/content/80/5/1031.long | Cell specific gene sets |
| Giri_etal_JLB2006_HIV_GALT | 16940334 | http://www.jleukbio.org/content/80/5/1031.long | Cell specific gene sets |
| Giri_etal_JLB2006_HIV_latInfCells | 16940334 | http://www.jleukbio.org/content/80/5/1031.long | Cell specific gene sets |
| Giri_etal_JLB2006_HIV_MDDC | 16940334 | http://www.jleukbio.org/content/80/5/1031.long | Cell specific gene sets |
| Giri_etal_JLB2006_HIV_NKcell | 16940334 | http://www.jleukbio.org/content/80/5/1031.long | Cell specific gene sets |
| Giri_etal_JLB2006_HIV_PBMC | 16940334 | http://www.jleukbio.org/content/80/5/1031.long | Cell specific gene sets |
| vant_Wout_2003_Cell_Division | 12502855 | http://ncbi.nlm.nih.gov/pubmed/12502855 | Functional gene sets |
| vant_Wout_2003_CellOrg_Defense | 12502855 | http://ncbi.nlm.nih.gov/pubmed/12502855 | Functional gene sets |
| vant_Wout_2003_GeneProt_Expr | 12502855 | http://ncbi.nlm.nih.gov/pubmed/12502855 | Functional gene sets |
| vant_Wout_2003_Metabolism | 12502855 | http://ncbi.nlm.nih.gov/pubmed/12502855 | Functional gene sets |
| vant_Wout_2003_SignalingComm | 12502855 | http://ncbi.nlm.nih.gov/pubmed/12502855 | Functional gene sets |
| vant_Wout_2003_StrucMobility | 12502855 | http://ncbi.nlm.nih.gov/pubmed/12502855 | Functional gene sets |
| vant_Wout_2003_Unknown | 12502855 | http://ncbi.nlm.nih.gov/pubmed/12502855 | Functional gene sets |
| vant_Wout_2003_UP | 12502855 | http://ncbi.nlm.nih.gov/pubmed/12502855 | total (all gene sets combined) down |
| vant_Wout_2003_DN | 12502855 | http://ncbi.nlm.nih.gov/pubmed/12502855 | total (all gene sets combined) up |
| Li_etal_HIV1_stages_JImm2009_ACUTE_UP | 19596987 | http://www.jimmunol.org/content/183/3/1975 | Acute HIV1 stage up |
| Li_etal_HIV1_stages_JImm2009_ACUTE_DN | 19596987 | http://www.jimmunol.org/content/183/3/1975 | Acute HIV1 stage down |
| Li_etal_HIV1_stages_JImm2009_ASYMPT_UP | 19596987 | http://www.jimmunol.org/content/183/3/1975 | Asymptomatic HIV1 stage up |
| Li_etal_HIV1_stages_JImm2009_ASYMPT_DN | 19596987 | http://www.jimmunol.org/content/183/3/1975 | Asymptomatic HIV1 stage down |
| Li_etal_HIV1_stages_JImm2009_AIDS_UP | 19596987 | http://www.jimmunol.org/content/183/3/1975 | AIDS stage up |
| Li_etal_HIV1_stages_JImm2009_AIDS_DN | 19596987 | http://www.jimmunol.org/content/183/3/1975 | AIDS stage down |
| Wu_etal_DiffReg_HIV_HCV_VirJ2015_HHvsCTR_UP | 25623235 | http://www.virologyj.com/content/12/1/4 | HIV/HCV co-infection vs. control up |
| Wu_etal_DiffReg_HIV_HCV_VirJ2015_HHvsCTR_DN | 25623235 | http://www.virologyj.com/content/12/1/4 | HIV/HCV co-infection vs. control down |
| Wu_etal_DiffReg_HIV_HCV_VirJ2015_HIVvsCTR_UP | 25623235 | http://www.virologyj.com/content/12/1/4 | HIV vs. control up |
| Wu_etal_DiffReg_HIV_HCV_VirJ2015_HIVvsCTR_DN | 25623235 | http://www.virologyj.com/content/12/1/4 | HIV vs. control down |
| Wu_etal_DiffReg_HIV_HCV_VirJ2015_HIVvsHCV_UP | 25623235 | http://www.virologyj.com/content/12/1/4 | HIV vs. HCV up |
| Wu_etal_DiffReg_HIV_HCV_VirJ2015_HIVvsHCV_DN | 25623235 | http://www.virologyj.com/content/12/1/4 | HIV vs. HCV down |
| Wu_etal_HIV_viremic_patients_Vir2013_HVLvsCTR_UP | 23158100 | http://dx.doi.org/10.1016/j.virol.2012.10.026 | High viral load vs. control up |
| Wu_etal_HIV_viremic_patients_Vir2013_HVLvsCTR_DN | 23158100 | http://dx.doi.org/10.1016/j.virol.2012.10.026 | High viral load vs. control down |
| Wu_etal_HIV_viremic_patients_Vir2013_INTvsCTR_UP | 23158100 | http://dx.doi.org/10.1016/j.virol.2012.10.026 | Intermediate load vs. control up |
| Wu_etal_HIV_viremic_patients_Vir2013_INTvsCTR_DN | 23158100 | http://dx.doi.org/10.1016/j.virol.2012.10.026 | Intermediate load vs. control down |

**Supplementary file 1B. Enrichment of TSG and TDG in the publicly available datasets (containing genes differentially expressed during HIV infection) from Supplementary file 1A.** The 62 gene sets compiled in file 1A were used for this analysis. Although we have obtained several enriched datasets, only significant enrichment (FDR < 0.05) is reported. P-values have been Bonferroni corrected for multiple testing. The data in this table corresponds to the enrichment plot shown in Figure 1–figure supplement 10A.

| **Gene class** | **Gene set** | **PMID** | **Binomial**  **p-value** | **Enrichment** | **Genes** |
| --- | --- | --- | --- | --- | --- |
| TSG | vant_Wout_2003_UP | 12502855 | 2.13e-5 | 4.8 | ACTN4 ARHGDIB ATP11B CD53 CD69 CLCN3 CRY1 DECR1 FDPS FLI1 INSIG1 LRRFIP1 MSN SLA TGFBR2 |
|  | Wu_etal_DiffReg_HIV_HCV_VirJ2015 | 25623235 | 2.18e-2 | 1.6 | ACTR3 APBA2 ARHGAP9 ARPP19 ATP11B ATP2A3 CD164 CD47 CKLF CYTH1 DIAPH1 ELMO1 ERBB2IP ETS1 ETV6 FAM117A FLI1 GATAD1 GMFG GOLT1B H2AFY HMGB1 IL17RA INPP5D ITGA6 LCP2 LPP LRIG1 MID1IP1 MSN PAN3 RNASEH2B RNF44 SACM1L SAMSN1 SERINC5 SERP1 SLA STK17B TBL1XR1 TRAF5 UTRN ZBTB44 ZNF384 |
|  | vant_Wout_2003_SignalingComm |  | 2.66e-2 | 4.8 | CD53 CD69 CLCN3 CRY1 PTP4A2 SLA TGFBR2 |
|  | Munier_etal_Retrovir2005_HIV-1_Latency_U1_and_ACH-2_DN |  | 3.23e-2 | 6.8 | DOCK10 EVL FLI1 LCP2 MYB |
|  | Munier_etal_Retrovir2005_HIV-1_Latency_U1_UP |  | 4.3e-2 | 6.5 | ANXA1 CD69 PEX13 RAB8B SREBF1 |
| TDG | REACTOME_HOST_INTERACTIONS_OF_HIV_FACTORS |  | 1.13e-6 | 7.1 | AP2S1 HMGA1 LCK NPM1 PSMB3 PSMB4 PSMB5 PSMC2 PSMD1 PSMD8 PSMD9 RANGAP1 RCC1 |
|  | REACTOME_HIV_INFECTION |  | 3.59e-5 | 4.8 | AP2S1 HMGA1 LCK NPM1 POLR2C PSMB3 PSMB4 PSMB5 PSMC2 PSMD1 PSMD8 PSMD9 RANGAP1 RCC1 |

**Supplementary file 1C. Functional annotation of canonical pathways enriched at TSG.** The MSigDB canonical pathway collection (c2.cp) was used for enrichment analysis of TSG and TDG. Only significant enrichment (FDR < 0.05) is reported. P-values have been Bonferroni corrected for multiple testing. This table is associated with Figure 1–figure supplement 10B.

| MsigDB | Binomial p-value | Enrichment |  |
| --- | --- | --- | --- |
| PID_TCR_PATHWAY | 1.32E-04 | 8.6 | CD247 CD28 CD3D CD3G INPP5D ITK LCP2 PRKCB PRKCQ PTEN |
| REACTOME_TCR_SIGNALING | 2.39E-04 | 9.5 | CD247 CD3D CD3G EVL ITK LCP2 PIK3CA PRKCQ PTEN |
| BIOCARTA_CTLA4_PATHWAY | 8.21E-04 | 16.3 | CD247 CD28 CD3D CD3G ITK PIK3CA |
| BIOCARTA_PAR1_PATHWAY | 2.10E-03 | 10.8 | ARHGEF10 ARHGEF2 ARHGEF7 GNA12 PIK3CA PLCB1 PRKCB |
| PID_RAC1_REG_PATHWAY | 2.53E-03 | 10.5 | ARHGAP9 ARHGEF2 ARHGEF7 ELMO1 PREX2 TIAM1 VAV3 |
| REACTOME_GENERATION_OF_SECOND_MESSENGER_MOLECULES | 4.09E-03 | 12.7 | CD247 CD3D CD3G EVL ITK LCP2 |
| KEGG_PHOSPHATIDYLINOSITOL_SIGNALING_SYSTEM | 4.61E-03 | 6.7 | CDS2 INPP4A INPP5D PIK3CA PIKFYVE PLCB1 PRKCB PTEN SYNJ2 |
| BIOCARTA_MYOSIN_PATHWAY | 9.63E-03 | 11.0 | ARHGEF10 ARHGEF2 ARHGEF7 GNA12 PLCB1 PRKCB |
| BIOCARTA_TOB1_PATHWAY | 1.79E-02 | 13.6 | CD247 CD28 CD3D CD3G TGFBR2 |
| BIOCARTA_TCAPOPTOSIS_PATHWAY | 1.94E-02 | 20.7 | CD247 CD28 CD3D CD3G |
| PID_CD8TCRPATHWAY | 2.48E-02 | 7.5 | CD247 CD28 CD3D CD3G LCP2 PRKCB PRKCQ |
| REACTOME_DOWNSTREAM_TCR_SIGNALING | 2.78E-02 | 9.2 | CD247 CD3D CD3G PIK3CA PRKCQ PTEN |

**Supplementary file 1D. Functional annotation of canonical pathways enriched at TDG.** The MSigDB canonical pathway collection (c2.cp) was used for enrichment analysis of TDG and TDG. Only significant enrichment (FDR < 0.05) is reported. P-values have been Bonferroni corrected for multiple testing. This table is associated with Figure 1–figure supplement 10B.

| MsigDB | Binomial p-value | Enrichment |  |
| --- | --- | --- | --- |
| REACTOME_METABOLISM_OF_RNA | 1.00E-08 | 5.4 | HSPA8 LSM4 PRMT5 PSMB3 PSMB4 PSMB5 PSMC2 PSMD1 PSMD8 PSMD9 RPL29 RPL35A RPL37A RPL38 RPL6 RPLP1 RPS12 RPS14 RPS19 RPS23 RPS5 RPS9 SNRPB |
| REACTOME_METABOLISM_OF_MRNA | 2.81E-08 | 5.7 | HSPA8 LSM4 PSMB3 PSMB4 PSMB5 PSMC2 PSMD1 PSMD8 PSMD9 RPL29 RPL35A RPL37A RPL38 RPL6 RPLP1 RPS12 RPS14 RPS19 RPS23 RPS5 RPS9 |
| REACTOME_HOST_INTERACTIONS_OF_HIV_FACTORS | 5.69E-06 | 7.6 | AP2S1 HMGA1 LCK NPM1 PSMB3 PSMB4 PSMB5 PSMC2 PSMD1 PSMD8 PSMD9 RANGAP1 RCC1 |
| KEGG_RIBOSOME | 6.57E-06 | 9.7 | RPL29 RPL35A RPL37A RPL38 RPL6 RPLP1 RPS12 RPS19 RPS23 RPS5 RPS9 |
| REACTOME_INFLUENZA_VIRAL_RNA_TRANSCRIPTION_AND_REPLICATION | 1.09E-04 | 5.9 | POLR2C RPL29 RPL35A RPL37A RPL38 RPL6 RPLP1 RPS12 RPS14 RPS19 RPS23 RPS5 RPS9 |
| REACTOME_INFLUENZA_LIFE_CYCLE | 1.40E-04 | 5.3 | CALR POLR2C RPL29 RPL35A RPL37A RPL38 RPL6 RPLP1 RPS12 RPS14 RPS19 RPS23 RPS5 RPS9 |
| REACTOME_DESTABILIZATION_OF_MRNA_BY_AUF1_HNRNP_D0 | 1.53E-04 | 11.7 | HSPA8 PSMB3 PSMB4 PSMB5 PSMC2 PSMD1 PSMD8 PSMD9 |
| REACTOME_3_UTR_MEDIATED_TRANSLATIONAL_REGULATION | 1.75E-04 | 5.7 | EIF3B RPL29 RPL35A RPL37A RPL38 RPL6 RPLP1 RPS12 RPS14 RPS19 RPS23 RPS5 RPS9 |
| REACTOME_HIV_INFECTION | 1.78E-04 | 5.2 | AP2S1 HMGA1 LCK NPM1 POLR2C PSMB3 PSMB4 PSMB5 PSMC2 PSMD1 PSMD8 PSMD9 RANGAP1 RCC1 |
| REACTOME_PEPTIDE_CHAIN_ELONGATION | 2.65E-04 | 6.1 | RPL29 RPL35A RPL37A RPL38 RPL6 RPLP1 RPS12 RPS14 RPS19 RPS23 RPS5 RPS9 |
| REACTOME_REGULATION_OF_APOPTOSIS | 3.13E-04 | 10.7 | DAPK1 PSMB3 PSMB4 PSMB5 PSMC2 PSMD1 PSMD8 PSMD9 |
| REACTOME_ER_PHAGOSOME_PATHWAY | 4.68E-04 | 10.1 | CALR PSMB3 PSMB4 PSMB5 PSMC2 PSMD1 PSMD8 PSMD9 |
| REACTOME_AUTODEGRADATION_OF_CDH1_BY_CDH1_APC_C | 6.81E-04 | 9.7 | ANAPC5 PSMB3 PSMB4 PSMB5 PSMC2 PSMD1 PSMD8 PSMD9 |
| REACTOME_SIGNALING_BY_WNT | 7.69E-04 | 9.5 | PPP2R5C PSMB3 PSMB4 PSMB5 PSMC2 PSMD1 PSMD8 PSMD9 |
| REACTOME_CROSS_PRESENTATION_OF_SOLUBLE_EXOGENOUS_ANTIGENS_ENDOSOMES | 1.10E-03 | 11.3 | PSMB3 PSMB4 PSMB5 PSMC2 PSMD1 PSMD8 PSMD9 |
| REACTOME_CDK_MEDIATED_PHOSPHORYLATION_AND_REMOVAL_OF_CDC6 | 1.10E-03 | 11.3 | PSMB3 PSMB4 PSMB5 PSMC2 PSMD1 PSMD8 PSMD9 |
| REACTOME_NONSENSE_MEDIATED_DECAY_ENHANCED_BY_THE_EXON_JUNCTION_COMPLEX | 1.18E-03 | 5.3 | RPL29 RPL35A RPL37A RPL38 RPL6 RPLP1 RPS12 RPS14 RPS19 RPS23 RPS5 RPS9 |
| REACTOME_REGULATION_OF_ORNITHINE_DECARBOXYLASE_ODC | 1.27E-03 | 11.0 | PSMB3 PSMB4 PSMB5 PSMC2 PSMD1 PSMD8 PSMD9 |

| REACTOME_SRP_DEPENDENT_COTRANSLATIONAL_PROTEIN_TARGETING_TO_MEMBRANE | 1.41E-03 | 5.2 | RPL29 RPL35A RPL37A RPL38 RPL6 RPLP1 RPS12 RPS14 RPS19 RPS23 RPS5 RPS9 |
| --- | --- | --- | --- |
| REACTOME_P53_INDEPENDENT_G1_S_DNA_DAMAGE_CHECKPOINT | 1.67E-03 | 10.6 | PSMB3 PSMB4 PSMB5 PSMC2 PSMD1 PSMD8 PSMD9 |
| REACTOME_AUTODEGRADATION_OF_THE_E3_UBIQUITIN_LIGASE_COP1 | 1.67E-03 | 10.6 | PSMB3 PSMB4 PSMB5 PSMC2 PSMD1 PSMD8 PSMD9 |
| REACTOME_SCF_BETA_TRCP_MEDIATED_DEGRADATION_OF_EMI1 | 1.67E-03 | 10.6 | PSMB3 PSMB4 PSMB5 PSMC2 PSMD1 PSMD8 PSMD9 |
| REACTOME_APC_C_CDH1_MEDIATED_DEGRADATION_OF_CDC20_AND_OTHER_APC_C_CDH1_TARGETED_PROTEINS_IN_LATE_MITOSIS_EARLY_G1 | 1.69E-03 | 8.6 | ANAPC5 PSMB3 PSMB4 PSMB5 PSMC2 PSMD1 PSMD8 PSMD9 |
| REACTOME_APC_C_CDC20_MEDIATED_DEGRADATION_OF_MITOTIC_PROTEINS | 1.88E-03 | 8.5 | ANAPC5 PSMB3 PSMB4 PSMB5 PSMC2 PSMD1 PSMD8 PSMD9 |
| REACTOME_VIF_MEDIATED_DEGRADATION_OF_APOBEC3G | 1.91E-03 | 10.4 | PSMB3 PSMB4 PSMB5 PSMC2 PSMD1 PSMD8 PSMD9 |
| REACTOME_TRANSLATION | 2.39E-03 | 4.5 | EIF3B RPL29 RPL35A RPL37A RPL38 RPL6 RPLP1 RPS12 RPS14 RPS19 RPS23 RPS5 RPS9 |
| REACTOME_ANTIGEN_PROCESSING_CROSS_PRESENTATION | 2.55E-03 | 8.1 | CALR PSMB3 PSMB4 PSMB5 PSMC2 PSMD1 PSMD8 PSMD9 |
| REACTOME_CDT1_ASSOCIATION_WITH_THE_CDC6_ORC_ORIGIN_COMPLEX | 3.18E-03 | 9.7 | PSMB3 PSMB4 PSMB5 PSMC2 PSMD1 PSMD8 PSMD9 |
| REACTOME_SCFSKP2_MEDIATED_DEGRADATION_OF_P27_P21 | 3.18E-03 | 9.7 | PSMB3 PSMB4 PSMB5 PSMC2 PSMD1 PSMD8 PSMD9 |
| REACTOME_P53_DEPENDENT_G1_DNA_DAMAGE_RESPONSE | 3.58E-03 | 9.5 | PSMB3 PSMB4 PSMB5 PSMC2 PSMD1 PSMD8 PSMD9 |
| REACTOME_REGULATION_OF_MRNA_STABILITY_BY_PROTEINS_THAT_BIND_AU_RICH_ELEMENTS | 5.42E-03 | 7.4 | HSPA8 PSMB3 PSMB4 PSMB5 PSMC2 PSMD1 PSMD8 PSMD9 |
| REACTOME_REGULATION_OF_MITOTIC_CELL_CYCLE | 5.88E-03 | 7.3 | ANAPC5 PSMB3 PSMB4 PSMB5 PSMC2 PSMD1 PSMD8 PSMD9 |
| REACTOME_ACTIVATION_OF_NF_KAPPAB_IN_B_CELLS | 7.82E-03 | 8.5 | PSMB3 PSMB4 PSMB5 PSMC2 PSMD1 PSMD8 PSMD9 |
| REACTOME_CYCLIN_E_ASSOCIATED_EVENTS_DURING_G1_S_TRANSITION_ | 8.66E-03 | 8.3 | PSMB3 PSMB4 PSMB5 PSMC2 PSMD1 PSMD8 PSMD9 |
| REACTOME_ASSEMBLY_OF_THE_PRE_REPLICATIVE_COMPLEX | 8.66E-03 | 8.3 | PSMB3 PSMB4 PSMB5 PSMC2 PSMD1 PSMD8 PSMD9 |
| REACTOME_ORC1_REMOVAL_FROM_CHROMATIN | 1.06E-02 | 8.1 | PSMB3 PSMB4 PSMB5 PSMC2 PSMD1 PSMD8 PSMD9 |
| BIOCARTA_PROTEASOME_PATHWAY | 1.19E-02 | 13.8 | PSMB3 PSMB4 PSMB5 PSMC2 PSMD8 |
| KEGG_PROTEASOME | 1.49E-02 | 9.7 | PSMB3 PSMB4 PSMB5 PSMC2 PSMD1 PSMD8 |
| REACTOME_FORMATION_OF_THE_TERNARY_COMPLEX_AND_SUBSEQUENTLY_THE_43S_COMPLEX | 2.04E-02 | 7.3 | EIF3B RPS12 RPS14 RPS19 RPS23 RPS5 RPS9 |
| REACTOME_M_G1_TRANSITION | 3.65E-02 | 6.7 | PSMB3 PSMB4 PSMB5 PSMC2 PSMD1 PSMD8 PSMD9 |
| REACTOME_MRNA_SPLICING | 4.10E-02 | 5.6 | HNRNPC PCBP2 POLR2C SF3A3 SF3B5 SNRNP200 SNRNP70 SNRPB |
| REACTOME_ACTIVATION_OF_THE_MRNA_UPON_BINDING_OF_THE_CAP_BINDING_COMPLEX_AND_EIFS_AND_SUBSEQUENT_BINDING_TO_43S | 4.61E-02 | 6.4 | EIF3B RPS12 RPS14 RPS19 RPS23 RPS5 RPS9 |
| REACTOME_METABOLISM_OF_PROTEINS | 4.77E-02 | 2.7 | ATP5G1 CALR EIF3B EIF5A RPL29 RPL35A RPL37A RPL38 RPL6 RPLP1 RPS12 RPS14 RPS19 RPS23 RPS5 RPS9 TBCD TIMM44 |
